# Influences of Combined Organic Fouling and Inorganic Scaling on Flux and Fouling Behaviors in Forward Osmosis

**DOI:** 10.3390/membranes10060115

**Published:** 2020-06-02

**Authors:** Youngpil Chun, Kwanho Jeong, Kyung Hwa Cho

**Affiliations:** 1 International Environmental Research Institute, Gwangju Institute of Science and Technology, Gwangju 61005, Korea; 2 School of Urban and Environmental Engineering, Ulsan National Institute of Science and Technology, Ulsan 44919, Korea; khcho@unist.ac.kr

**Keywords:** forward osmosis, organic fouling, gypsum scaling, combined fouling

## Abstract

This study investigated the influence of combined organic fouling and inorganic scaling on the flux and fouling behaviors of thin-film composite (TFC) forward osmosis (FO) membranes. Two organic macromolecules, namely, bovine serum albumin (BSA) and sodium alginate (SA), and gypsum (GS), as an inorganic scaling agent, were selected as model foulants. It was found that GS scaling alone caused the most severe flux decline. When a mixture of organic and inorganic foulants was employed, the flux decline was retarded, compared with when the filtration was performed with only the inorganic scaling agent (GS). The early onset of the conditioning layer formation, which was due to the organics, was probably the underlying mechanism for this inhibitory phenomenon, which had suppressed the deposition and growth of the GS crystals. Although the combined fouling resulted in less flux decline, compared with GS scaling alone, the concoction of SA and GS resulted in more fouling and flux decline, compared with the mixture of BSA and GS. This was because of the carboxyl acidity of the alginate, which attracted calcium ions and formed an intermolecular bridge.

## 1. Introduction

During the last decade, forward osmosis (FO) was extensively researched in various areas with more than 1500 referred publications [1,2]. However, because it is energy inefficient when hybridized with other membrane processes, such as reverse osmosis (RO), especially for the treatment of low salinity target solutions, researchers are investigating other desalination and dewatering processes where RO need not be employed [3,4]. Processes exposed to high fouling/scaling environments, such as brine treatment and in bioreactor applications, are good examples [5,6,7]. Thus, the challenges in controlling fouling in FO are significant because more complex feed waters are prevalent in various water sources [8].

The study of both inorganic scaling agents and organic macromolecules is crucial, as both types of foulants are ubiquitous in natural waters [9,10]. It has been demonstrated that combined fouling exhibits different behaviors and involves different mechanisms, compared with single fouling [10]. Arkhangelsky et al. [11] reported that flux decline by the combined effect of multicomponent foulants, including either alginate and bovine serum albumin (BSA) or silica nanoparticles (SiO_2_) and alginate, were severe, whereas the flux decline by the combination of BSA and SiO_2_ were negligible. Another synergetic effect of combined fouling by alginate and silica colloids was reported by Kim et al. [12]. In this study, the overall flux decline by combined fouling was greater than the sum of the individual contributions of each foulant. Moreover, the types of foulant also affected flux reversibility. The reversibility of the alginate–silica fouling was affected by the synergistic effect, while the individual organic or colloidal fouling was completely reversible. In the aforementioned studies, the feed water chemistry (presence of calcium (Ca) ions) and hydrodynamic (osmotic pressure (OP), cross-flow velocity, and pH) conditions considerably affected the flux and fouling profiles.

There have been arguments on whether surface or bulk crystallization is prevalent in the scaling mechanism [9,13]. It is now widely recognized that scaling is a combination of both, depending on the saturation concentrations; operating conditions, such as the transmembrane pressure, cross-flow velocity, and permeate flux [13]; or surface topology, and chemistry [14]. Benecke et al. [9] revealed that severe concentration polarization (CP) initiated gypsum (GS) surface (heterogeneous) crystallization rather than bulk (homogeneous) crystallization. The coexisting organic macromolecules, namely BSA and humic acid (HA), significantly retarded the flux decline, while alginate exacerbated the flux decline. Liu and Mi also claimed that an enhanced CP was responsible for the accelerated heterogeneous crystallization of GS [15]. It has been reported that when organic macromolecules are present in feed water, inhibitory anti-scaling or aggravated scaling effects and change in the size and morphology of GS crystals are observed. This is because of the property of the organic macromolecules, particularly the surface chemical functionality and their affinity to scaling agents [9,10,11].

Although considerable efforts have been devoted to investigating fouling behaviors in FO processes, the influence of combined fouling or fouling precursors on natural water sources is still not completely understood. Here, we reported the effects of the coexistence of organic macromolecules and inorganic scaling agents on the FO process. Two commercially available FO membranes were utilized in the treatment of model foulants, and their flux profiles and fouling densities were examined. Notably, newly available commercial FO membrane was utilized in this study, and its properties were compared with those of a conventional FO membrane.

## 2. Materials and Methods

### 2.1. Feed Solution (FS), Draw Solution (DS), and Membranes

Because of their abundance in natural waters, sodium alginate (alginic acid, SA) and BSA were selected as the representatives of protein and natural organic matter (NOM) fouling precursors, respectively, and GS (CaSO_4_·2H_2_O) was selected as an inorganic scaling agent [15,16,17]. To accelerate the rates of fouling, FSs with high fouling potentials were prepared, utilizing 200 mg L^−1^ of either SA or BSA. In the case of GS, a saturation index of 1.3 was prepared, in which 35 mM (3.88 g L^−1^) calcium chloride (CaCl_2_), 20 mM (3.28 g L^−1^) sodium phosphate (Na_2_SO_4_), and 19 mM (1.11 g L^−1^) sodium chloride (NaCl) were dissolved in MilliQ water (Millipore™, Bedford, MA, USA). Moreover, NaCl was utilized as a draw solute. All the chemicals were obtained from Sigma Aldrich, St. Louis, MO, USA. Two commercial polyamide (PA)-based thin-film composite (TFC)-FO membranes were purchased from Hydration Technology Inc. (HTI, Albany, OR, USA) and Toray Chemical (Seoul, Korea), and they were denoted as TFC-1 and TFC-2, respectively. The membrane samples were stored in MilliQ water, at 4 °C for ≥24 h before use.

### 2.2. FO System Configuration and Filtration Experiments

A custom-made bench-scale FO system was employed with minor modification from a previous study [18], to perform the filtration experiments. The system included a cross-flow membrane cell with two symmetric flow channels; 88 mm long, 42 mm wide, and 2 mm deep. Diamond-shaped spacers were utilized on both sides of the membrane cell, for mechanical support. FS and DS were pumped in countercurrent directions, at flow rates of 1000 mL min^−1^ (cross-flow velocity = 9.5 cm s^−1^) in each channel on both sides of the membrane, employing gear pumps (WT3000-1JB, Longer Pump, Baoding, China). The FS tank was placed on a balance (KERN FKB, KERN & SOHN GmbH, Balingen, Germany) to determine the water flux $\left(J_{w}\right)$ by measuring the weight variations during the experiments. The conductivity was also measured with a conductivity meter (Orion Star A212, Thermo Scientific, Beverly, MA, USA).

For the filtration experiments, each pristine membrane was stabilized by filtering 2 L of MilliQ water as the feed and 2 L of DS, for 30 min. Thereafter, the following model foulants were introduced into the FS tank: GS, a mixture of BSA + GS, and a mixture of SA + GS. The initial flux was set at the identical value of 20 L m^−2^ h^−1^ (LMH) as a benchmark to eliminate the effect of the differences in the initial flux [19]. To obtain the identical initial flux (20 LMH), different concentrations of DS were utilized where 4 M and 0.8 M NaCl were used for TFC-1 and TFC-2, respectively. The baseline experiment was also performed under identical experimental conditions to demonstrate the effects of fouling on the flux decline. The filtration duration was 20 h, during which FS and DS were continuously concentrated and diluted, respectively. All the filtration experiments were performed in the active layer (AL) facing FS (AL-FS; FO mode), and the results were obtained from the duplicates, within 2.5% of the difference in the initial flux.

### 2.3. Structural and Transport Parameters of the Membrane

A single FO experimental method was employed to determine the transport and structural parameters of the osmotic membranes. The experimental method for this membrane characterization can be found in the literature [20]. In this study, the single-stage method was performed based on our previous study [21], which was optimized from the aforementioned study.

### 2.4. Membrane Surface and Fouling Characterizations

The surface topology of the membranes was characterized by field-emission scanning electron microscopy (FESEM; JEM-7200F, JEOL, Tokyo, Japan). Energy-dispersive X-ray spectroscopy (EDX) analysis was also performed with an EDX spectrometer (EDAX Genesis, JEOL, Japan), coupled with an EDX software. The surface charge of the membrane was measured, employing a SurPass streaming potential analyzer (SurPass™ 3, Anton Paar GmbH, Graz, Austria). The streaming potential were measured in a background electrolyte (10 mM KCl), over a pH range of 3 to 9 at ambient temperature (25 °C). The contact angles were measured with a contact angle goniometer (Contact Angle System OCA, DataPhysics Instruments, Filderstadt, Germany), by the sessile drop method. At least 10 measurements were obtained for each sample. The surface roughness of the membrane was determined by atomic force microscopy (AFM; PSIA NX 10, Park Systems, Suwon, Korea). The samples were observed in a 5 × 5 µm^2^ area, using a non-contact cantilever (NCHR 10M, Park Systems, Korea). The arithmetic average roughness (R_a_) of the membrane surface was computed with the associated software. The Attenuated total reflectance (ATR)–Fourier transform infrared (FTIR) spectra were obtained using an FTIR spectrometer (IR Prestige-21, Shimadzu, Tokyo, Japan), equipped with an ATR element. The spectra from 600 to 4000 cm^−1^ were scanned. All the spectra were baseline-corrected using ambient air.

## 3. Results and Discussion

### 3.1. FO Membrane Characteristics

The surface characteristics of the membrane are summarized in Table 1. The surface charges between the two samples were comparable. TFC-2 was less hydrophilic on both AL and the support layer (SL), but it still possessed a much smoother surface than TFC-1, as revealed by the contact angle and AFM measurements. The surface hydrophilicity and roughness were probably the vital parameters that affect the flux and fouling behaviors of an FO membrane [22]. The significance of these parameters was greater in the early stages of fouling and in short-term regime [23], while their influences diminished in a prolonged investigation [24].

TFC-2 has a greater water permeability (*A*) value than TFC-1 while the salt permeance (*B*) value was also substantially higher. *S* value is generally defined by Equation (1) [20]:

(1) S=tτ/ε,

where t is the thickness of SL, τ is the tortuosity, and ε is the porosity. The *S* value for TFC-2 was 220 µm, compared with 600 µm obtained for TFC-1. Considering the higher hydrophobicity of TFC-2, which affects the diffusion of water and solute molecules [25] as revealed by the contact angle of SL, the low membrane thickness (87 µm) induced a smaller *S*–value for TFC-2, as τ is difficult to control [26].

Additionally, the fabrication of an FO membrane with a high *A/B* ratio is crucial to prevent the loss of draw solutes. A higher *A/B* ratio will induce the sustainability of DS and reduce fouling and the scaling potential of FS [27]. Although TFC-2 exhibited remarkable *A*, the low *A/B* ratio was due to their high *B*. $J_{w}$ and *S* could be optimized, but it is likely at the expense of higher reverse salt diffusion (RSD) and the loss of mechanical strength [27,28].

### 3.2. Flux Profiles during Membrane Fouling

**Figure 1:**
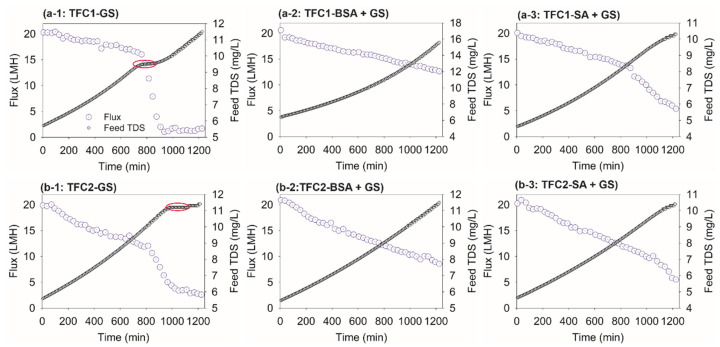
Permeate water flux, $J_{w},$ patterns and the corresponding total dissolved solids (TDS) concentration of FS: (**a**) TFC-1, (**b**) TFC-2, (1) GS, (2) BSA + GS, and (3) SA + GS.

The concentration of the total dissolved solids (TDS) of FS was compared with that of the $J_{w}$ patterns in Figure 1. It is interesting to note that when the onset of an acute flux decline initiated during the filtration of GS, the feed TDS stabilized for a certain period (highlighted with red circles) and thereafter increased again, as shown in Figure 1a-1,b-1. It was assumed that the surface crystallization was initiated at that point, and GS crystals started to grow and cover the membrane surface. It is known that an adequate induction time was required for the initiation of GS scaling. Additionally, before the onset of scaling, the membrane surface was relatively free of large crystals [29]. In our study, the severe flux decline was observed from the 10th h, for TFC-1, and the 15th h, for TFC-2. In the case of combined SA and GS fouling, substantial flux declines were observed at the end of the experiment for both samples. However, the relaxation and sudden elevation of the feed TDS were not observed, as evidently in the case of GS scaling. This also implies that although the membrane surface was fouled, AL was neither completely blocked nor did the GS surface crystallization occur. This was confirmed by the membrane surface FESEM images (to be discussed in Section 3.3). 

**Figure 2:**
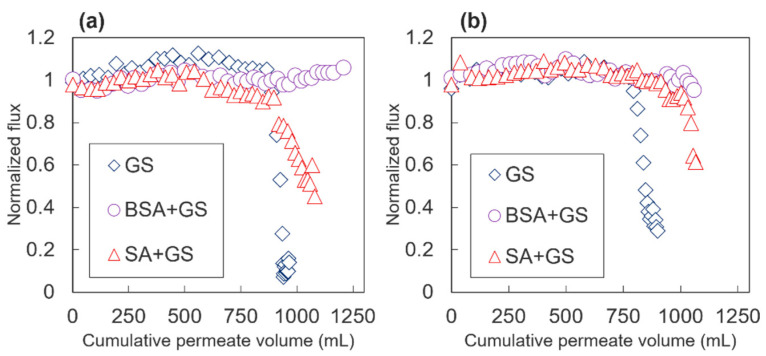
Permeate $J_{w}$ of (**a**) TFC-1 and (**b**) TFC-2 during fouling and scaling experiments. The experiments were conducted for 20 h. The permeate $J_{w}$ was normalized by the pure $J_{w}$ utilizing MilliQ water as the feed and 1 M NaCl as DS, at the same initial flux of 20 ± 0.5 LMH.

The normalized flux patterns obtained from each filtration experiment are shown in Figure 2. Severe flux declines were observed during GS scaling in both samples. It is generally known that when alginate and GS (or calcium ions) coexist, the flux declines were accelerated in the FO membranes [10,15,30]. Although a more severe flux decline was observed during the combined fouling of SA + GS than that during the fouling of BSA + GS, these mixtures did not have any severe synergetic effects, compared with the flux during GS scaling alone. When concoctions of BSA + GS or SA + GS were utilized, the flux declines were retarded, compared with when GS scaling alone was utilized. A sharper decline in $J_{w}$ due to the mixture of SA + GS, than in the concoction of BSA + GS, was observed for both membranes. Additionally, 55% and 39% $J_{w}$ declines were observed for TFC-1 and TFC-2 by the SA + GS mixtures, respectively. This was attributed to the “intermolecular bridging” mechanism between the alginate molecules and calcium ions, resulting in the formation of a cross-linked alginate gel layer on the membrane surface [31]. This thick gel layer afforded resistance to $J_{w}$, as well as augmented the cake-enhanced OP (CEOP) or cake-enhanced CP (CECP), resulting in elevated OP or CP near the membrane surface on the feed region [15,32,33,34]. This eventually accelerated more severe flux declines. In the FO processes, CEOP or CECP could also be exacerbated because of RSD [21,35]. The alginate layer could also be observed with the naked eye at the end of the SA + GS filtration experiments (photos not shown). The high density of the carboxyl functional groups in the alginate fouling layers attracted calcium ions and accelerated GS heterogeneous crystallization, while the inhibition of GS surface scaling by BSA was due to the low carboxylic acidity and steric obstruction [15,36]. A previous study revealed that in the course of the GS scaling experiments, calcium ions were absorbed into the Aldrich humic acid (AHA) and alginate, but not on the BSA conditioning layer [15]. It was also found that the BSA conditioning layer was rigid and unaffected by the calcium ions from GS while the alginate layer became increasingly rigid, after the adsorption of the calcium ions. The rigidity was likely due to the formation of the calcium-alginate complex. Combined BSA + GS fouling was insignificant in both samples; there was no flux decline in the TFC-1, while only a 5% flux decline was observed for TFC-2.

The alginate molecules act as nuclei in GS crystal growth, significantly increasing the size of the GS crystals and accelerating the crystallization kinetics [10]. Either combined GS and alginate fouling, or pre-coated alginate and GS scaling was dominated by heterogeneous crystallization rather than by homogeneous crystallization. Here, the sizes of the deposited GS crystals in the combined fouling experiment were smaller and much sparser than those in GS scaling alone (this will be discussed in the following subsection). The organic macromolecule adsorption was likely the key mechanism of this occurrence. That is, the organic macromolecule first formed a conditioning layer [18], after which this layer prevented the surface crystallization of GS and retarded the acute flux decline. Consequently, the overall flux declines were deterred during the combined BSA + GS and SA + GS fouling experiments, compared with during GS scaling experiment alone.

### 3.3. Analyses of the Membrane Foulants

**Figure 3:**
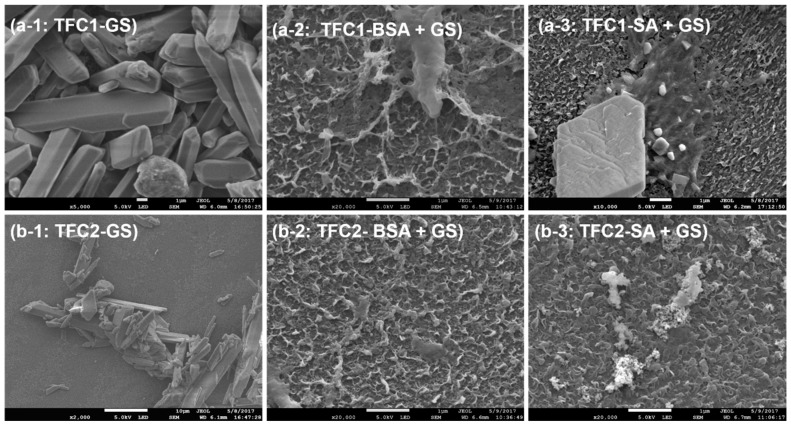
Field-emission scanning electron microscopy (FESEM) images of (**a**) TFC-1 and (**b**) TFC-2 after (1) GS, (2) BSA + GS and (3) SA + GS scaling or foulings, respectively.

The fouling density was identified by FESEM–EDX and ATR–FTIR. It was observed that the surface of TFC-1 was completely covered by the GS crystals, as shown in Figure 3a-1. It was also observed that the surface of TFC-1 was completely blocked by GS, based on the results from FESEM-EDX (Table 2). Carbon is the main component of the pristine TFC matrix. However, only a weak peak, which accounted only 9.77 ± 0.26 atomic percentage (at.%) of the total elemental composition, after GS scaling in TFC-1, was observed, compared with 85.70 ± 0.10 at.% present in the pristine membrane. Conversely, sparsely deposited GS crystals were observed on TFC-2, as shown in Figure 3b-1, and a much weaker peak of Ca (2.39 ± 0.02 at.%) was observed, compared with that observed in TFC-1 (11.48 ± 0.02 at.%) in spite of a substantial flux decline after GS scaling. The lower scaling propensity of TFC-2 attributed to the smooth membrane surface of AL although TFC-1 was marginally more hydrophilic than TFC-2 (Table 1). Further, high RSD of TFC-2 could limit some extents of GS scaling [37]. Although the combined SA + GS fouling resulted in significant flux declines, it was observed that they inhibited the surface crystallization of GS scaling, as proven by FESEM-EDX results, both presented in Figure 3 and Table 2. GS crystals were barely observed, while gel-like substances, which were likely derived from the alginate gel layers, were more prevalent, as shown in Figure 3a-3,b-3, compared with Figure 3a-1,b-1, where they were barely observed. Specifically, Ca was substantially reduced during combined fouling, in comparison with that during GS scaling alone as exhibited in Table 2.

**Figure 4:**
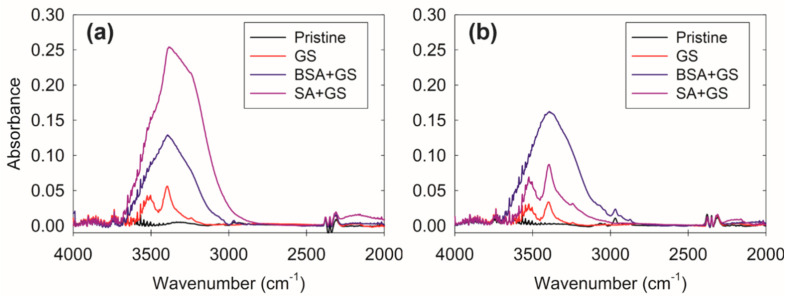
Representative attenuated total reflectance (ATR)-Fourier transform infrared (ATR-FTIR) absorbance spectra for (**a**) TFC-1 and (**b**) TFC-2.

The ATR-FTIR spectra were also employed as a qualitative tool to reveal GS scaling on the membrane surface (Figure 4). Both AL and SL could be detected because of the penetration depth of the ATR-FTIR is greater than 300 nm in the wavenumbers lower than 2000 cm^−1^. Moreover, ATR-FTIR is more surface-sensitive in the high wavenumber regions, with a penetration depth of 200 nm in the range of 4000 to 2600 cm^−1^ [14]. Thus, we focused on the wavenumbers greater than 2000 cm^−1^ in this study. A broad peak at 3400 cm^−1^ (O–H stretching) and a sharp peak at 2970 cm^−1^ (C–H stretching) correspond to the carboxyl functional groups on the membrane surface [38]. However, the broad peak at 3400 cm^−1^ was not observed in the pristine samples. Nevertheless, after the GS scaling experiments, the peaks appeared at 3400 cm^−1^, indicating that there were specific interactions between Ca ions and the carboxylic functional groups of GS and TFC membrane surface, respectively in both cases. The ratio $I_{3400}/I_{2970}$ could also be employed as an indication of the specific interaction in the focused spectra. The ratios were 57.6 for TFC-1, and 36.6 for TFC-2, respectively. The formation of calcium carboxylate on the membrane surface resulted in O–H stretching, which could represent the calcium concentration on the membrane surface. This eventually initiated the formation of GS pre-nucleation crystals and subsequent GS surface crystallization [14]. Further, the broad absorption peak at 3300–3500 cm^−1^ also indicated the presence of protein (N–H stretching vibration, at 3265 cm^−1^) and polysaccharide-like substances (O–H vibration, at 3412 cm^−1^) in the fouling layer [39,40,41]. These masked peaks were significant in the case of SA + GS and BSA + GS fouling experiments. This could be explained by the combined fouling of organic and inorganic constituents.

Previous studies have discovered the effects of combined fouling on membrane processes, including FO [10,11,15], pressure-retarded osmosis (PRO) [37], nanofiltration (NF) [9], and RO [42]. The underlying mechanism for their synergetic or inhibitory effects on the flux and fouling behaviors vary, depending on the chemistry of the foulants and membrane surface and the foulant-foulant interactions. Although most previous studies demonstrated the exacerbated effect of combined fouling on filtration processes, the present study revealed that combined fouling hinders the increase in GS scaling because of the coexistence of the organic macromolecules in FS. There is, therefore, a need for further investigation of the effect of combined fouling on FO processes. The flux behaviors, due to combined fouling, are governed by the chemistry of the feed water and the membrane surface, foulant properties and characteristics, and permeate flux and hydrodynamic shear. Thus, the investigation of the differences in the effects of combined fouling on membrane processes utilizing different driving forces (i.e., pressure-driven and osmotically-driven membrane processes (ODMP)) should be considered. In that regard, either response surface methodology (RSM) based on the design of experiments (DOE) or artificial intelligence (AI) techniques such as machine learning could facilitate more effective analysis on the relationship between diverse influential factors and their interactions on the combined fouling [43]. Furthermore, regardless of whether the effects of combined fouling on membrane performance are positive or not, fouling is still an essential factor to consider during ODMPs. FO possesses an outstanding advantage in niche areas where the regeneration of DS is not required or in the treatment of challenging feed waters [44]. Thus, anti-fouling membrane development or membrane surface modification, for fouling mitigation, must be considered [45]. Although many researchers have attempted to develop such membranes, the practicality is still limited to lab-scale, and further investigations are required to overcome these challenges.

## 4. Conclusions

This study investigated the influences of combined fouling, by organic macromolecules and inorganic GS scaling, on the FO process. Two commercial FO membranes were investigated and compared in this regard. The results suggested that TFC-2 was more tolerant of inorganic scaling than TFC-1, in terms of fouling density, although substantial flux declines were observed in both samples. In the case of TFC-1, the atomic elemental composition (at.%) of Ca and the peak intensity value, at certain wavenumbers ($I_{3400}/I_{2970}$) were 11.48% and 57.6, respectively, while in that of TFC-2, they were 2.39% and 36.6, as revealed by FESEM-EDX and ATR-FTIR, respectively. Combined GS + BSA or GS + SA fouling resulted in less severe flux declines and scaling deposition, compared with that of GS scaling alone. Flux declines were retarded by the earlier onset of a conditioning layer formation by the organic macromolecules. The mixture of GS and SA accelerated the flux decline compared with the concoction of GS and BSA because of the high density of carboxyl acid functional group on the alginate surface. Our findings suggest that combined fouling did not necessarily exacerbate the flux decline in all cases and that flux decline also depended on the membrane intrinsic properties, surface chemistry, and foulant-foulant interactions.

## Figures and Tables

**Table 1:** Membrane surface characteristics. AL: active layer; SL: support layer. Results for TFC-1 were adapted from our previous study [21].

Membrane ID	Thickness (μm)	Contact Angle (°)	Surface Charge ^a^ (mV)	Roughness ^b^ (nm)	Pure Water Permeability, *A* (L m^−2^ h^−1^) (LMH)/bar)	Salt Permeability, *B* (LMH)	*A*/*B* (bar^−1^)	Structural Parameter, (μm)	Error (%)	$\mathrm{Water}\;\mathrm{Flux},\;J_{w}\;(\mathrm{LMH}){\;}^{c}$	$\mathrm{Reverse}\;\mathrm{Salt}\;\mathrm{Flux},\;J_{s}\;(g\;m^{-2}\;h^{-1}\;(\mathrm{gMH})){\;}^{c}$
TFC-1	115	45.5 ± 5.0 (AL) 56.0 ± 2.5 (SL)	−20.9 ± 2.5	41.7 ± 1.5	3.6	1.6	2.3	600	6.65	14.3 ± 1.8	5.4 ± 1.5
TFC-2	87	49.1 ± 2.4 (AL) 68.6 ± 1.4 (SL)	−19.3 ± 1.3	24.9 ± 2.4	11.0	5.9	1.9	220	8.87	33.3 ± 1.8	20.2 ± 1.1

^a^ Zeta potential values, at pH 7, ^b^ Arithmetic average surface roughness (R_a_), ^c^ $J_{w}$ and $J_{s}$ were measured, utilizing MilliQ water as the feed solution (FS) and 1 M NaCl as the draw solute at 1000 mL min^−1^ cross-flow rate, in the AL-FS orientation.

**Table 2:** Elemental compositions (at.%) of pristine and fouled membrane samples by FESEM-energy-dispersive X-ray spectroscopy (EDX) analysis.

Membrane ID	Type of Foulant	C	O	S	Na	Cl	Ca
TFC-1	Pristine	85.70 ± 0.10	11.93 ± 0.11	2.37 ± 0.02	- *	-	-
	GS	9.77 ± 0.26	65.98 ± 0.25	12.04 ± 0.02	0.38 ± 0.02	0.35 ± 0.01	11.48 ± 0.02
	BSA + GS	76.30 ± 0.16	11.21 ± 0.44	2.01 ± 0.25	4.79 ± 0.28	4.98 ± 0.08	0.72 ± 0.18
	SA + GS	63.53 ± 0.13	12.57 ± 0.07	4.39 ± 0.01	6.78 ± 0.05	11.03 ± 0.06	1.69 ± 0.04
TFC-2	Pristine	86.12 ± 0.08	11.13 ± 0.06	2.75 ± 0.03	-	-	-
	GS	65.79 ± 0.17	25.69 ± 0.16	4.37 ± 0.02	0.82 ± 0.01	0.93 ± 0.01	2.39 ± 0.02
	BSA + GS	72.39 ± 0.19	12.85 ± 0.07	1.93 ± 0.38	5.89 ± 0.05	6.72 ± 0.16	0.22 ± 0.03
	SA + GS	74.87 ± 0.07	18.77 ± 0.05	2.91 ±0.16	0.92 ±0.05	1.20 ± 0.14	1.33 ± 0.05

* Not detected.
